# Association between Current Substance Use, Healthy Behaviors, and Depression among Ukrainian College Students

**DOI:** 10.3390/ijerph21050586

**Published:** 2024-05-02

**Authors:** Julia Burlaka, Renee M. Johnson, Christina N. Marsack-Topolewski, Kathryn Hughesdon, Jill Owczarzak, Oleksii Serdiuk, Roman Bogdanov, Viktor Burlaka

**Affiliations:** 1 Department of Mental Health, Johns Hopkins Bloomberg School of Public Health, Baltimore, MD 21205-1996, USA; rjohnson@jhu.edu; 2 School of Social Work, Eastern Michigan University, Ypsilanti, MI 48197, USA; ctopole1@emich.edu; 3 School of Nursing, Eastern Michigan University, Ypsilanti, MI 48197, USA; kabramos@emich.edu; 4 Department of Health, Behavior & Society, Johns Hopkins Bloomberg School of Public Health, Baltimore, MD 21205-1996, USA; jillowczarzak@jhu.edu; 5 Research Lab for Psychological Support of Law Enforcement, Kharkiv National University of Internal Affairs, 61080 Kharkiv, Ukraine; serdyuk@univd.edu.ua (O.S.); bogdanov.roman@univd.edu.ua (R.B.); 6 School of Social Work, Wayne State University, Detroit, MI 48202, USA

**Keywords:** depression, substance use, college students, mental health, health-promoting behaviors, Ukraine, psychological, coping

## Abstract

Depression is a pervasive issue among college students worldwide, yet there is a significant gap in the literature regarding its prevalence and coping strategies in the Ukrainian context. The present study aims to fill this gap by investigating how substance use and health-promoting behaviors relate to depressive symptoms among Ukrainian college students. Health-promoting behaviors are an important strategy to prevent depression, whereas substance use can contribute to depression or make it harder to manage. Given the substantial psychosocial problems and stressors related to the ongoing war in Ukraine and the limited availability of mental health services for college students, it is important to understand how we can encourage college students to keep themselves mentally healthy. A cross-sectional study was conducted among college students on 10 campuses in 2018. Almost 16% of participants met a cut-off for depression. Female students and those who were older reported significantly higher depressive symptoms. Participants were more likely to report depressive symptoms if they were more often involved with alcohol and cannabis use, were older, and engaged in fewer health-promoting behaviors. Tobacco use was not significantly associated with depressive symptoms. Our findings suggest a moderate prevalence of depressive symptoms in our study population. We recommend implementing balanced public health interventions that address risk factors (such as substance use) while also promoting protective behaviors and can be tailored to the specific cultural and environmental context of the region.

## 1. Introduction

Depression is a widely recognized public health issue among college students globally. The prevalence of depression among university students varies around the world; it is estimated to be 24% in low- and middle-income countries [1], 41% in the U.S. [2], 23.8% in China [3], 18.4% in Spain [4], and varies from 15 to 32% in Ukraine [5,6,7]. Depression is particularly harmful for college students, as it jeopardizes their likelihood of success in school and further career opportunities. Depressive symptoms such as disturbed sleep, low energy levels, difficulty concentrating, or suicidal thoughts are associated with poor academic productivity [8,9,10,11,12]. Depressed college students may encounter problems recalling material and a reduced ability to perform daily tasks (e.g., self-care and housework), along with missing social activities available on campus. The negative impact of depression during college may result in lifelong consequences, such as poor academic success, being unable to fulfill requirements for career opportunities, developing suicidal ideation, and impairments in professional and social spheres of life [13,14,15,16,17].

The lifetime prevalence of nicotine, cannabis (which was illegal in Ukraine at the time this study was conducted), and alcohol among Ukrainian college students is estimated at 60%, 19%, and 80%, respectively, as measured before February 2022 [18,19]. For college students, substance use can worsen depression, adversely impacting school performance and decreasing the likelihood of successfully earning a degree. Substance use is a maladaptive coping strategy to manage stress, and many college students report substance use as a coping strategy [20,21,22,23,24,25,26,27]. Depressive disorders commonly co-occur with alcohol, smoking, and cannabis use [24]. Consistent with the self-medication hypothesis, individuals with depression may use substances to cope with mental health symptoms [28]. Prior, smaller-scale research indicated that a lack of substantial mental health services led Ukrainian college students to similarly use substances as a coping mechanism for their psychological distress [29,30]. In a study examining the comorbidity between mental disorders and nicotine dependence, depressive disorders predicted the first onset of regular smoking and the progression from regular smoking to dependence [31,32]. Because of the interrelationship between substance use and depression among college students around the world, it is critical to examine substance use in association with depression.

In contrast to substance use as a maladaptive coping strategy, health-promoting behaviors are positive ways to manage stress. Health-promoting behaviors can prevent depression, reduce its severity, and promote recovery. Health-promoting behaviors may include physical activity, healthy stress management, and life appreciation (defined as an individual’s efforts to like themselves, to feel happy and content, to think positively, and to feel interested and challenged every day), which generally improve psychological well-being and reduce depressive symptoms [33,34,35,36,37]. Research from several countries suggests that college students who went through stress management intervention programs reported significantly less depression than their peers who did not attend such programs [35]. Research on the effectiveness of these health-promoting behaviors in alleviating depressive symptoms among college students is lacking in Ukraine.

Given the high levels of depression among college students and its negative consequences for academic, professional, and social life, it is important to understand factors associated with this condition. Much of what we know about factors associated with depression among college students comes from studies of students in the US and Western Europe. Thus, it is necessary to generate new knowledge about substance use and health-promoting behaviors in association with depression among Ukrainian college students. 

Health-promoting behaviors are an important strategy to prevent depression, whereas substance use can contribute to depression or make it harder to manage. However, little is known about the utilization of health-promoting behaviors by Ukrainian college students. The purpose of the current study is to characterize how depressive symptomatology is associated with substance use and health-promoting behaviors among Ukrainian college students. Based on previous research showing linkages between depression and substance use, we expect to find that students engaged in risky use of cigarettes, alcohol, and cannabis are more likely to experience depression. We also expect to find that health-promoting behaviors are protective against depression. Given substantial social problems and stressors related to growing up in a social turbulence environment (e.g., poverty and high crime rates in the 90’s, the Orange Revolution of 2004, the annexation of Crimea, and conflict in Eastern Ukraine in 2014), limited access to mental health services, it is important to understand how we can encourage college students to keep themselves mentally healthy. This study aims to contribute to the literature on the role of health-promoting behaviors, substance use, and depression among Ukrainian college students. The present research is particularly important because Ukrainian college students are not inclined to use professional psychological services for various reasons (e.g., stigma, lack of trust towards mental health care providers, confidentiality concerns) [30]. Instead, they often rely on self-coping strategies (i.e., taking a walk, reading a book, engaging in physical activity, using alcohol or weed, or smoking) [29].

## 2. Materials and Methods

### 2.1. Participants and Data Collection

Data for this investigation come from the Ukrainian Student Success Study (USSS), with data collection occurring between March and December of 2018. As part of USSS, college students across 10 campuses in Ukraine were recruited via announcements that described the goals and procedures of the study. Interested individuals contacted trained faculty members or research assistants in their respective colleges for further guidance on participating in the study. The cooperation rate for this study was 90%, as calculated by dividing the number of actual participants by the number of students in participating universities. All data collection sessions were carried out with small groups of participants on university premises and proctored by trained research assistants. The study was approved by the Committee of Ethics and Deontology of the Ukrainian National Academy of Medical Sciences Institute of Neurology, Psychiatry, and Narcology. 

Before engaging in the study activities, each participant signed an informed consent form. Participants were then provided a link to an electronic survey, and they used their personal devices, such as tablets or laptops, to provide their responses, which were captured through Qualtrics software (https://www.qualtrics.com, accessed on 31 January 2024). College computers were made available to those study participants who needed them. All data collected through Qualtrics software was stored on a secure online platform until it was later accessed for analysis purposes. The electronic survey gathered information on student demographics as well as on overall mental health, childhood experiences, health-promoting behaviors, and substance use.

### 2.2. Measures

Use of tobacco, alcohol, and cannabis was measured with the Alcohol, Smoking and Substance Involvement Screening Test (ASSIST), which inquired about the use of alcohol, tobacco, and cannabis in the past three months. Responses to questions regarding the urge to use and problems related to using substances or failing to do what is expected because of substance use ranged from Never, Once or Twice, Monthly, Weekly, and Daily/Almost daily. Responses were rated on a 0–6, 0–7, or 0–8 scale, depending on the question. For example, the urge to use question was rated on a 0 to 6 scale (0 = Never, 3 = Once or Twice, 4 = Monthly, 5 = Weekly, 6 = Daily/Almost daily), and the question asking about problems related to substance use was rated on a 0 to 7 scale (0 = Never, 4 = Once or Twice, 5 = Monthly, 6 = Weekly, 7 = Daily/Almost daily). Questions asking if others expressed concern about your substance use and if you tried to cut down on substance use and failed had the following response options: 0 = Never; 6 = Yes, in the past three months; 3 = Yes, but not in the past three months. Scores from these questions were added together across each individual substance to produce risk score for each substance [38]. The scores from 0–3 for cannabis and tobacco and from 0–10 for alcohol indicate lower risk. A score between 4 and 26 for cannabis and tobacco and 11 and 26 for alcohol indicates a moderate risk of health and other problems and suggests that a person may already have experienced some of the problems. A score of 27 or above for any substance suggests substance dependence or a high risk of developing one [38]. The ASSIST tool, a well-established and validated measure of high-risk substance use, has previously been employed in Ukraine.

Engagement in healthy behaviors was assessed with the Adolescent Health-promoting Scale (AHP-SF), a 21-item instrument measuring nutrition, social support, health responsibility, life appreciation, exercise habits, and stress management [39]. The reported Cronbach’s alpha for this scale was 0.91 and 0.86 [39,40]. The responses ranged from Never = 1 to Always = 5 points. Items are summed to generate a total score for health-promoting behaviors, ranging from 21 to 105. Higher score is indicative of better health-promoting behaviors. In this study, the alpha coefficient was 0.91.

Depressive symptoms were measured using the 10-item Center for the Epidemiological Studies of Depression Short Form (CES-D) [41,42]. Participants reported how often they felt depressive symptoms within the last week (e.g., “I had trouble keeping my mind on what I was doing”). Two items asked about positive affect (e.g., “I was happy”, “I felt hopeful about the future”). Each item was rated on a 4-point Likert scale: 1 = rarely or none of the time (<1 day) to 4 = most or all of the time (5–7 days). Prior to analysis, the items were scored from 0 to 3, and the two positive affect items were reverse scored. Higher scores were indicative of more depressive symptoms. The alpha coefficient was 0.84.

### 2.3. Data Analyses

The sample characteristics were analyzed using mean and standard deviation for continuous variables and frequency and percentage for categorical ones. Bivariate analyses included calculating Pearson’s correlation coefficient and *t*-test. ANOVA was performed to determine if there was a statistically significant difference in the mean value of depressive scores across three age categories, followed by post-hoc pairwise comparisons (Tukey’s test). To assess collinearity, preliminary assessment of the correlation matrix of substance use risk scores was conducted with correlation values equal to 0.3, 0.4, and 0.4, which did not reveal any correlations exceeding the critical value of 0.8. Stepwise multiple linear regression analysis was used to examine the association between tobacco, alcohol, and cannabis use, health-promoting behaviors, and depression (outcome variable), controlling for gender and age. All analyses were conducted using Stata version 16 [43].

## 3. Results

A total of 997 college students (68.5% women and 31.5% men) took part in this investigation. Age ranged from 17 to 24, with an average of 19.06 ± 1.6 years. The majority of participants were not employed (75%) and never married (89.5%), as shown in Table 1.

Almost 16% of participants met a cut-off for depression (a score of 10 or higher). Female students and those who were older reported significantly higher depressive symptoms (Table 2). Eight percent (*n* = 62) of participants scored at a moderate risk level for tobacco use, and 20% scored at a moderate risk for alcohol use, indicating that participants may be experiencing some of the problems related to the use of these substances now and that a brief intervention might be appropriate [38]. Few participants (1%, *n* = 6) scored at high risk for alcohol involvement, suggesting a high risk of alcohol dependency or being dependent and possibly experiencing varied problems related to alcohol use and may benefit from being referred to specialized assessment and treatment [38]. All participants scored at lower risk for cannabis use. The mean score on health-promoting behaviors was 68.1 (SD = 14.1). Intercorrelations between study variables are presented in Table 3.

The stepwise multiple regression was conducted with three blocks of variables. The first block included age and gender as the predictors, with depressive symptomology as the dependent variable. In block two, substance use (tobacco, alcohol, and cannabis use) was also included as a predictor variable, and health-promoting behaviors were added in block three (Table 4). 

Overall, the results suggested that the first model was significant *F*(3, 912) = 11.71, *p* < 0.001, *R*^2^ = 0.04. Both age and gender were significantly associated with depressive symptoms (Table 4). The second model (*F*(6, 764) = 15.43, *p* < 0.001, *R*^2^ = 0.11) that included substance use variables showed significant improvement from the first model (*∆F*(3,764) = 18.81, *p* < 0.001, *∆R*^2^ = 0.071). It suggested that students were more likely to report depressive symptoms if they were more often involved with alcohol (*β* = 0.20, *p* < 0.001) and cannabis use (*β* = 0.09, *p* < 0.05), and were in two older age categories (*β* = 0.10, *p* < 0.01; *β* = 0.09, *p* < 0.05). The third model (*F*(7, 758) = 22.31, *p* < 0.001, *R*^2^ = 0.17) included health-promoting behaviors and showed significant improvement from the second model (*∆F*(1, 758) = 56.91, *p <* 0.001, *∆R*^2^ = 0.06) with results suggesting that students were more likely to report depressive symptoms if they were more often involved with alcohol (*β* = 0.17, *p* < 0.001) and cannabis use (*β* = 0.11, *p* < 0.01), were older (*β* = 0.09, *p* < 0.01; *β* = 0.09, *p* < 0.05) and engaged in fewer health-promoting behaviors (*β* = −0.25, *p* < 0.001).

Participants’ gender was statistically associated with depressive symptoms only in the first model. Overall, when age and gender were included in the model, the variables explained 4% of the variance. When tobacco, alcohol, and cannabis use variables were added to the second model, it explained 11% of the variance. In the final model, including health-promoting behaviors accounted for 17% of the variance. Tobacco use was not significantly associated with depressive symptoms.

We then tested for effect modification. No effect modification was found based on participants’ age groups or gender, indicating no significant difference in the relationship between health-promoting behaviors and the depressive symptoms score based on the participants’ gender or age.

## 4. Discussion

Depressive symptoms in college students are associated with poor academic productivity and can have a negative impact on their academic lives [13,16,17]. In this sample of Ukrainian college students, nearly 16% met the cut-off criteria for depression, which is consistent with results from previous studies [5,6,7]. Therefore, it is important to identify factors that are associated with depressive symptoms in college students to identify targets for interventions and improve academic productivity. 

Results indicated that female students and those who were older reported significantly higher depressive symptoms. This aligns with previous research suggesting that women are disproportionately affected by depression compared with men [44]. Various theories have been proposed to explain this gender disparity, ranging from hormonal fluctuations to psychosocial factors [45,46]. The intersectionality of gender with other social determinants of health could provide additional insights into these findings. The association between age and depressive symptoms in the study sample might suggest that older students face unique challenges that contribute to higher depressive symptoms. These could include pressures from nearing graduation, job search anxiety, or balancing academic obligations with external responsibilities such as employment or family care [47]. It is also possible that older students might have a higher level of self-awareness regarding their emotional state, leading to more accurate self-reporting of depressive symptoms compared with younger students. A negative correlation was observed between health-promoting behaviors and the use of alcohol and tobacco. This relationship may be attributed to a heightened awareness of the health risks associated with the consumption of these substances, which influences individuals to either abstain or moderate their use. Furthermore, those who prioritize health-promoting behaviors could also harbor negative attitudes towards harmful habits and perceive social pressures that discourage the use of alcohol and tobacco. Further qualitative research is warranted to explore the underlying motivations among Ukrainian college students who engage in health-promoting behaviors while avoiding alcohol and tobacco.

The results of this study indicate that Ukrainian college students were more likely to report depressive symptoms if they engaged in alcohol or cannabis use. Previous studies support a similar association in that psychological risk factors (e.g., depression) were commonly associated with alcohol, cannabis, and tobacco use [23]. In studies with college students, the presence of depressive symptoms was related to alcohol problems [21]. Moreover, research indicates that depressive disorders commonly co-occur with alcohol, smoking, and cannabis use [24]. Both alcohol and smoking can serve as a means of coping with stress and negative emotions [48,49,50]. Magee and Connell found in a longitudinal study of adolescents and young adults that substance use coping was a predictive factor in later depression diagnoses [51]. Given these findings, students could be using these substances as a way to cope with their depressive symptoms. Identifying more positive coping strategies with these students could reduce substance use rates and improve depressive symptoms.

While previous research suggests a co-occurrence between smoking and depressive symptoms and that tobacco use can be a predictive factor of greater depression among young adults [23,24], tobacco use was not associated with depressive symptoms among participants in this study. One possible explanation is that there was not much heterogeneity in terms of tobacco usage in our sample, and therefore we did not observe the relationship. 

Students who engaged in fewer health-promoting behaviors had higher depression scores. Previous research supports these findings. In a longitudinal study examining the influence of physical activity (PA) on the trajectory of depression from adolescence through emerging adulthood, Mcphie and Rawana found that physical activity was a protective factor against depression [52]. In another study, Bailey and colleagues conducted a meta-analysis of randomized controlled trials that delivered various types of PA interventions to adolescents and emerging adults [37]. The findings from the sixteen studies that were analyzed supported that participants in the intervention groups had lower levels of depression compared with those in control conditions. These results indicate that PA may be an effective tool for decreasing depression symptoms in college students. Implementing interventions focused on PA in Ukrainian college students could be beneficial in reducing their depression symptoms.

Other health-promoting behaviors have also been associated with lower depression scores. In a study with Chinese undergraduate students, Wang and colleagues found that social support moderated the relationship between stress and depression [53]. In another study, social support from family and friends was negatively associated with depressive symptoms in a college sample [54]. Another concept associated with lower depression scores is stress management. In a study with Canadian university students, Sawatzky and colleagues found that stress management self-efficacy partially mediated the relationship between stress and depression [55]. Life appreciation is another concept that was measured in the health-promoting behavior questions. Although previous research specifically examining the concept of life appreciation in depression is minimal, similar concepts are associated with lower depression scores. One question in the life appreciation category asks about purpose in life. In a meta-analysis that examined the relationship between purpose in life and depression and anxiety, Boreham and Schutte found that purpose in life was associated with lower levels of depression [56]. Focusing on promoting these health behaviors could be another strategy to target in interventions to improve depressive symptoms.

This study identified a number of factors associated with depression symptoms in Ukrainian college students. Findings highlight the importance of fostering health-promoting behaviors in students. Given the impact of depression on college students, identifying interventions to improve depression symptoms is important. Additionally, with rising mental health conditions in Ukraine due to the ongoing war [57], it is even more imperative to implement strategies to reduce these symptoms. Although substance use and health-promoting behaviors are associated with depression, targeting these concepts together in an intervention could be more beneficial than targeting each concept individually. In a multimodal online lifestyle intervention for depression in an adult sample, those who participated in the intervention had significantly lower depression scores post-intervention compared with the waitlist control group [58]. The multimodal intervention focused on education about moderate alcohol consumption, a healthy diet, increased physical activity, and stress reduction through mindfulness-based strategies and meditation. Future research would benefit from studying a multimodal intervention that educates on substance use, sleep hygiene, and health-promoting behaviors in college students. 

There are a number of strengths and limitations to this study. The present study uses a large sample of Ukrainian college students to examine concurrent individual lifestyle behaviors and their associations with depressive symptoms, and it is the first study with this population to do so. Additionally, this study measures the levels of depressive symptoms in college students after the start of the conflict in the eastern part of Ukraine in 2014 and prior to February 2022 [59]. These results need to be interpreted in light of some limitations. First, the study employs a cross-sectional design, which makes it difficult to establish causality. Next, the study includes a sample of students from only ten out of the hundreds of Ukrainian universities. It should also be noted that depression may have been a risk factor for substance use for participants in the current study. Future research in this region should focus on examining the interplay between substance use, mental health, and health-promoting behaviors in college students using a longitudinal design. Despite some limitations, our findings provide a crucial baseline against which changes in student behavior due to recent events can be measured. They serve as a historical snapshot that can inform longitudinal studies seeking to understand the impact of events such as epidemics or conflicts on student wellbeing. Moreover, although external circumstances have transformed since the data were collected, the underlying psychological processes and responses to stressors, as captured by the findings, are likely to remain relevant. The findings of this study offer insights into the coping mechanisms that students might resort to when faced with adversity. Given the contextual changes, future research should investigate the current situation of Ukrainian college students and focus on analyzing how recent events may have altered or intensified the patterns we observed. The findings underscore important associations that warrant further investigation to improve depressive symptoms in Ukrainian college students. Furthermore, these findings have the potential to inform balanced public health interventions that address risk factors while also promoting protective behaviors and can be tailored to the specific cultural and environmental context of the region.

## 5. Conclusions

We found a moderate prevalence of depressive symptoms in our study population. Female students and those who were older reported significantly higher depressive symptoms. We investigated the risk and health-promoting behaviors associated with depressive symptoms. Participants were more likely to report depressive symptoms if they were more often involved with alcohol and cannabis use, were older, and engaged in fewer health-promoting behaviors. We did not observe that tobacco use was significantly associated with depressive symptoms, although this association has been reported in other studies. Our findings suggest that future interventions with Ukrainian college students should focus on maladaptive and positive coping strategies and encouragement to adopt health-promoting behaviors to support their mental well-being. 

## Figures and Tables

**Table 1:** Demographic characteristics of study participants (*n* = 997).

Variables		*n* (%)
Age	Mean ± SD	19.06 ± 1.6
Age Category	17–19 years	664 (66.6%)
	20–21 years	242 (24.3%)
	22–24 years	91 (9.1%)
Sex	Male	314 (31.5%)
	Female	683 (68.5%)
Program of Study	Social Work	286 (29.0%)
	Medical School	126 (12.0%)
	Pedagogy	94 (9.4%)
	Psychology	388 (39.0%)
	Educational Therapy	103 (10.3%)
Employment Status	Employed	180 (18.1%)
	Not Employed	748 (75.0%)
	Missing	69 (6.9%)
Relationship Status	Single, Never Married	892 (89.5%)
	All Other	105 (10.5%)
Region	Central	285 (28.6%)
	Western	260 (26.1%)
	Eastern	433 (43.4%)
	Southern	19 (1.9%)
Ethnicity/Nationality	Ukrainian	962 (96.5%)
	Russian	15 (1.5%)
	All Other	20 (2.0%)

**Table 2:** Mean scores (standard deviation) on the CESD *, by demographic characteristics (*n* = 997).

	M	SD	*p*
Age Category			<0.001
17–19 years	7.32	4.86	
20–21 years	9.15	5.37	
22–24 years	9.28	5.45	
Sex			<0.001
Male	7.00	4.70	
Female	8.36	5.24	

* M = mean score on the CESD, SD = standard deviation, *p* = statistical significance of the overall group difference (ANOVA and *t*-test were used to obtain *p*-values). Post hoc pairwise comparisons using Tukey’s HSD test indicated significant mean differences between age categories. Category 2 differed from Category 1 by 1.83 units (*p* < 0.05; 95% CI [0.89, 2.77]). Category 3 differed from Category 1 by 1.95 units (*p* < 0.05; 95% CI [0.54, 3.37]). No significant difference was found between Categories 3 and 2 (*p* > 0.05; 95% CI [−1.43, 1.68]).

**Table 3:** Mean scores, Cronbach’s Alpha Coefficients, and Pearson Correlation Coefficients among Mental and Behavioral Health Measures.

Variable ^1^	M	SD	Cronbach’s Alpha	1	2	3	4	5
1 Depression (CESD)	7.9	5.1	0.84	-				
2 Tobacco use risk score (ASSIST)	6.1	8.7	0.84	0.15 ***	-			
3 Alcohol use risk score (ASSIST)	6.1	6.3	0.73	0.28 ***	0.44 ***	-		
4 Cannabis use risk score (ASSIST)	0.1	0.4	0.63	0.18 ***	0.33 ***	0.36 ***	-	
5 Health-promoting behaviors (AHPS)	68.1	14.1	0.91	−0.30 ***	−0.14 ***	−0.17 ***	−0.01	-

*** *p* < 0.001. **^1^** CESD: Center for the Epidemiological Studies of Depression. ASSIST: Alcohol, Smoking and Substance Involvement Screening Test. AHPS: Adolescent Health-Promoting Scale.

**Table 4:** Regression Coefficients of Age, Sex, Substance Use, and Health-Promoting Behaviors on Depressive Symptomology.

Predictor	Model 1			Model 2			Model 3		
	*B*	*β*	SE	*B*	*β*	SE	*B*	*β*	SE
Age category 20–21 years ^a^	1.58 ***	0.13	0.41	1.26 **	0.10	0.43	1.13 *	0.09	0.42
Age category 22–24 years	1.81 **	0.09	0.60	1.73 *	0.09	0.62	1.63 *	0.09	0.60
Sex	−1.00 *	−0.09	0.36	−0.73	−0.06	0.42	−0.66	−0.06	0.41
Tobacco use risk score (ASSIST)				0.03	0.05	0.02	0.01	0.02	0.02
Alcohol use risk score (ASSIST)				0.17 ***	0.20	0.03	0.14 ***	0.17	0.03
Cannabis use risk score (ASSIST)				1.36 *	0.09	0.55	1.61 **	0.11	0.53
Health-promoting behaviors score (AHPS)							−0.10 ***	−0.25	0
*Intercept*	7.72			6.55			13.45		
*R* ^2^	0.04			0.11			0.17		
*∆R* ^2^				0.07 ***			0.06 ***		
*F*	11.71			15.43			22.31		

^a^ Estimates are from a multiple regression model with three models; *B* = unstandardized regression coefficient, SE = standard error of *B*, *β* = standardized coefficient. Reference groups for categorical variables: age category 17–19; female participants. * *p* < 0.05. ** *p* < 0.01.*** *p* < 0.001.

## Data Availability

The raw data supporting the conclusions of this article will be made available by the authors on request.
